# Helminthological Survey of the European Brown Hare (*Lepus europaeus*) in Türkiye, with A Note on Haplotype and Network Analyses of *Dicrocoelium dendriticum* and *Passalurus ambiguus*

**DOI:** 10.1007/s11686-026-01278-1

**Published:** 2026-04-21

**Authors:** Kadir Kalkan, Ufuk Erol, Omer Faruk Sahin, Husnu Furkan Sakar, Kursat Altay

**Affiliations:** 1 Veterinary Department Laboratory Technician and Veterinary Health Program, Şarkışla Aşık Veysel Vocational School, Sivas Cumhuriyet University, Sivas, Turkey; 2 Department of Parasitology, Faculty of Veterinary Medicine, Sivas Cumhuriyet University, Sivas, Turkey; 3 Department of Veterinary Parasitology, Institute of Health Sciences, Sivas Cumhuriyet University, Sivas, Turkey

**Keywords:** European brown hare, Helminth, DNA sequence analysis, Haplotype and network analyses, Phylogenetic analyses, Türkiye

## Abstract

**Purpose:**

Several studies have been conducted to determine the prevalence and presence of helminth species in European brown hare populations in various parts of its geographical range. However, there are limited data on helminth species circulating among European brown hare populations in Türkiye. The aim of this study is to investigate the presence and prevalence of helminth species in European brown hares in Türkiye, to perform phylogenetic analyses of the identified species, and to determine haplotype diversity and network properties of *Dicrocoelium dendriticum* and *Passalurus ambiguus.*

**Methods:**

Internal organs from 109 European brown hares were collected and examined for the presence of helminth species. The helminths detected were identified based on their morphological properties. DNA extraction was performed from a randomly selected sample of each species, and the *COI* gene region was amplified by PCR. Bidirectional DNA sequence analysis was done, and the obtained nucleotide sequences were uploaded to GenBank. Phylogenetic analyses of helminth species were performed, and their genetic characteristics were revealed. Additionally, haplotype diversity and network properties of *D. dendriticum* and *P. ambiguus* were determined.

**Results:**

It has been determined that 70.64% (77/109) of animals were infected with at least one helminth species. While 35.78% (39/109) of the animals were infected with one helminth species, 34.84% (38/109) were infected with two or more helminth species. Six helminth species, *Dicrocoelium dendriticum*,* Andrya rhopalocephala*,* Mosgovoyia pectinata*,* Trichuris leporis*,* Passalurus ambiguus*, and *Nematodirus leporis*, were identified. *Andrya rhopalocephala* was detected for the first time in Türkiye. Phylogenetic analyses of the *COI* gene of these helminth species, except for *T. leporis*, were performed, and all obtained nucleotide sequences were deposited to GenBank. Furthermore, haplotype and network analyses of *D. dendriticum* and *P. ambiguus* isolates were done, and it was determined that 11 *D. dendriticum* haplotypes and five *P. ambiguus* haplotypes were found to be circulating among hosts in different parts of the world.

**Conclusion:**

This study is the first to perform microscopic and molecular detection of helminth species in European brown hares in Türkiye. The study determined that helminth prevalence is quite high in this animal in the country. It is considered that the data obtained within the aim of the study will contribute to understanding the epidemiology and genetic characteristics of helminth species circulating in wild animals.

**Supplementary Information:**

The online version contains supplementary material available at 10.1007/s11686-026-01278-1.

## Introduction

The European brown hare (*Lepus europaeus*) belongs to the family Leporidae, and the order Lagomorpha [1]. This species is distributed throughout almost all countries in the European continent, as well as in Iran, Iraq, and Russia. It is regarded as a game animal in many parts of the world [1, 2]. The European hare is a primary food source for many predator species (fox, wolf, lynx, predatory birds, and snakes). This animal contributes to the dispersal of seeds of many plant species in nature and also plays a role in maintaining plant diversity in the ecosystem by affecting the growth and structure of the vegetation. The above-mentioned data indicated that the European hare is one of the important animals of the healthy terrestrial ecosystem [2–5].

The European hare can live in various habitats, from sea level to high Arctic tundra, and population densities range from two to 275 individuals in 100 hectares [2]. However, its population size has declined since the beginning of the 20th century. According to the IUCN report, this species is considered “Least Concern” in some European countries. In some countries, like Norway, Austria, Germany, and Switzerland, this decline in population has led to the species being classified as “near threatened” or “threatened” [1, 2]. Reasons such as expanding human settlement, invasive agricultural practices, hunting activities, predator animals, climate change, and diseases, have been associated with the population decline of the European hare [1–3, 5, 6].

Studies have revealed that the European brown hare may be exposed to several bacterial, viral, and parasitic pathogens [1, 2, 6–8]. While some of these pathogens are specific to hares, others can cause serious infections not only in hares but also in domestic animals and humans [1, 6, 9]. Furthermore, parasitic pathogens can cause significant declines in hare populations due to the clinical symptoms and deaths they cause [9–11]. Numerous studies have been conducted to identify helminth species that may cause infection among the European hare populations in various parts of the world [11–16]. However, in Türkiye, limited data are present on the presence and prevalence of helminth species in the European hare [17–21]. Furthermore, no studies have been conducted in the country to investigate helminth species in the European brown hare using molecular methods. This study aims: (i) to investigate the presence and prevalence of helminth species in the European hare in Türkiye, (ii) to determine the phylogenetic features of the identified helminth species using DNA sequence analyses, and (iii) to research haplotype diversity and network properties of *D. dendriticum* and *P. ambiguus*.

## Materials and Methods

### Sampling Area and Samples

The field studies were carried out in Sivas Province (35°50′–38°14′ E and 38°32′–40°16′ N, altitude 596–2922 m). A large part of the province is covered by high plateaus and mountains [22, 23]. The human population is low, and the landscape is quite wide, hilly, and rugged, resulting in a rich and diverse wildlife [24].

The study material consisted of internal organs obtained from 109 European brown hares. These animals were hunted between November 2024 and January 2025 by licensed hunters. No all internal organs could be obtained from each animals. We studied intestines (n: 92), liver (n: 91), spleen (n: 84), lung (n: 67), heart (n: 35), and kidney (n: 23) samples.

### Microscopic Examination of Internal Organs and Species Identification of Helminths

Intestines were examined using the sedimentation and counting technique following the methods described by Hofer et al. [25] and Bowman [26]. After examination, sediments from each organ were collected separately into beakers. All of these sediments were divided into 10 mL portions and examined under a stereomicroscope for the presence of helminths. All helminths were collected into 70% ethanol for further examination. The preliminary examinations of the identified helminths were performed under a light microscope. One specimen of each helminth species was set aside for molecular analysis, and these samples were stored at 4 °C for DNA extraction.

Cestodes were stained with borax-carmine, whereas trematodes and nematodes were cleared in lactophenol. The species identifications were done according to the morphological features of helminths [27–32].

### DNA Extraction and PCR Assay

Molecular analyses of the helminth species were performed to confirm microscopic identification and to conduct phylogenetic, haplotype, and network analyses. One randomly selected sample from each helminth species was used for DNA extraction. Helminth tissue (50 mg) was used for DNA extraction. Before the extraction process, helminth tissues were fragmented using a sterile scalpel and transferred to a 1.5 mL sterile microcentrifuge tube. DNA extraction was performed with PureLink™ Genomic DNA Mini Kit (Cat. No.: K182002, Invitrogen™), and all procedures were done according to the manufacturer’s instructions. After the DNA extraction process, the obtained DNA was measured with a NanoDrop (Denovix Ds-11, USA), and samples were stored at − 20 °C until the PCR assay.

The PCR assay was carried out using primers JB3 (5′-TTTTTTGGGCATCCTGAGGTTTAT-3′) and JB4.5 (5′-TAAAGAAAGAACATAATGAAAATG-3′) to target 446 bp parts of the *COI* gene region of helminth species [33]. The PCR master-mix was prepared in a total volume of 25 µL, including: 10 × EZ-FD Reaction buffer (Lot. No.: O807R0K, BioBasic), 1 µL (10 pmol/µL) of each of the primers, 200 µM of each dNTP (Lot.No.: R22256RoX, BioBasic), 1.25 U of EZ-FD PCR High Fidelity DNA Taq polymerase (2.5U/µL) (Lot.No.: Q6A00120KF, BioBasic), 2.5 µL template DNA, and DNase-RNase-free sterile water. The PCR procedure was performed as described in detail by Erol et al. [34]. The PCR products were loaded onto a 1% agarose gel and subjected to electrophoresis at 90 V for 60 min. After electrophoresis, the agarose gel was stained with ethidium bromide for 20 min and checked with a UV transilluminator for the specific amplicons.

### Phylogeny, Haplotype, and Network Analyses of Helminth Species

The DNA sequence analyses were performed for phylogenetic analyses of identified helminth species. For this purpose, bidirectional DNA sequence analyses were performed using primers JB3 and JB4.5 [33]. The sequence files were opened with FinchTV (1.4.0) (Geospiza Inc., Seattle, Washington, USA) to check the chromatogram qualities. Forward and reverse sequences were aligned using the MEGA-11 software [35], and consensus sequences were determined. These sequences were deposited in GenBank, and accession numbers were obtained.

Nucleotide similarities between helminth species were determined using the BLASTn algorithm, and the results were recorded. Moreover, the number of haplotypes (*H*), haplotype diversity (*Hd*), nucleotide diversity (*π*), the average number of nucleotide differences (*κt*), and neutrality tests were assessed with DnaSP v6 [36], and these data were extracted as nexus files for use in network analyses. Haplotype networks were constructed using the Minimum Spanning Network (MSN) algorithm in [37] in PopART (version 1.7) [38].

### Permissions

A permission to collect parasitological samples from hares was given by the Republic of Türkiye, Ministry of Agriculture and Forestry, General Directorate of Nature Conservation and National Parks (Date: 18.11.2024, Document number: E-21264211-288.04-16723783). Ethical permission was issued by the Sivas Cumhuriyet University Animal Experiments Local Ethics Committee (Date: 27.12.2024, Approval number: 56202830-050.01.01-114).

## Results

A total of 109 hares were examined for the presence of helminth species. Out of them 77 (70.64%) animals were infected with helminth species. At least one helminth species was detected in 81.52% (75/92) of the intestinal samples and 4.40% (4/91) of the liver samples. No helminth species were detected in spleen (n: 84), lung (n: 67), heart (n: 35), and kidney (n: 23) samples.

**Fig. 1:**
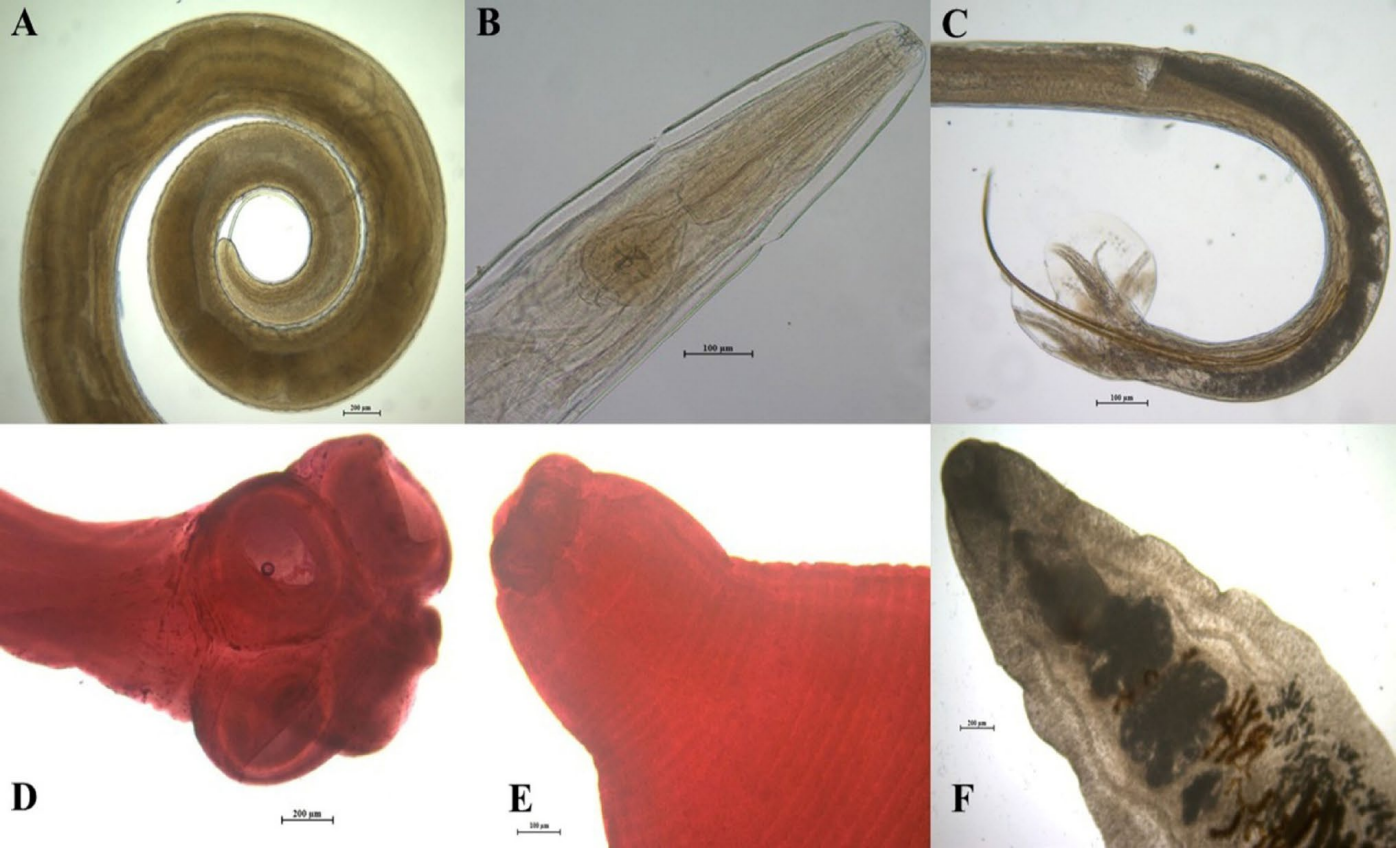
Figures of helminth species identified in this study. (**A**) Posterior part of *Trichuris leporis*, (**B**) Anterior part of *Passalurus ambiguus*, (**C**) Posterior part of *Nematodirus leporis*, (**D**) Scolex of *Andrya rhopalocephala*, (**E**) Scolex of *Mosgovoyia pectinata*, (**F**) Anterior part of *Dicrocoelium dendriticum*

Five helminth species were identified in the intestinal samples, whereas one helminth species was detected in liver samples. The intestinal helminth species were nematodes *Trichuris leporis* (Froelich, 1789) (Trichuridae), *Passalurus ambiguus* (Rudolphi, 1819) (Oxyuridae), and *Nematodirus leporis* (Chandler, 1924) (Trichostongylidae), as well as the anoplocephalid cestodes *Andrya rhopalocephala* (Riehm, 1881) and *Mosgovoyia pectinata* (Goeze, 1782) (Fig. 1). No trematode species were detected in the intestinal samples. In the liver samples, the trematode species *Dicrocoelium dendriticum* (Rudolphi, 1819) (Dicrocoeliidae) was detected (Fig. 1). The detailed information about prevalence, range, mean intensity, and mean abundance was presented in Table 1.

**Table 1:** Prevalence, range, mean intensity, and mean abundance of the helminth species

Species	Number of infected animals	Range of intensity	Mean intensity	Mean abundance
*Dicrocoelium dendriticum*	4	1–17	6.25	0.23
*Andrya rhopalocephala*	12	1–31	3.83	0.42
*Mosgovoyia pectinata*	2	1–4	2.50	0.05
*Trichuris leporis*	57	1–141	14.19	7.42
*Passalurus ambiguus*	34	1–951	249.73	77.89
*Nematodirus leporis*	25	1–37	6.00	1.38

A single helminth species was determined in 39 (35.78%) animals, whereas mixed-infections were seen in 38 (34.86%) animals, of which 21 animals had two helminth species, 15 animals had three helminth species, and two animals had four helminth species (Table 2).

**Table 2:** The type of mixed-infection in the study

Type of the mixed infection	Number of positive samples
*T. leporis* + *P. ambiguus*	9
*T. leporis* + *N. leporis*	5
*T. leporis* + *A. rhopalocephala*	3
*P. ambiguus* + *N. leporis*	2
*T. leporis* + *D. dendriticum*	1
*P. ambiguus* + *A. rhopalocephala*	1
*T. leporis* + *P. ambiguus* + *N. leporis*	8
*T. leporis* + *P. ambiguus* + *A. rhopalocephala*	4
*P. ambiguus* + *N. leporis* + *A. rhopalocephala*	2
*T. leporis* + *N. leporis* + *D. dendriticum*	1
*T. leporis* + *P. ambiguus* + *N. leporis* + *A. rhopalocephala*	2
Total	38

**Fig. 2:**
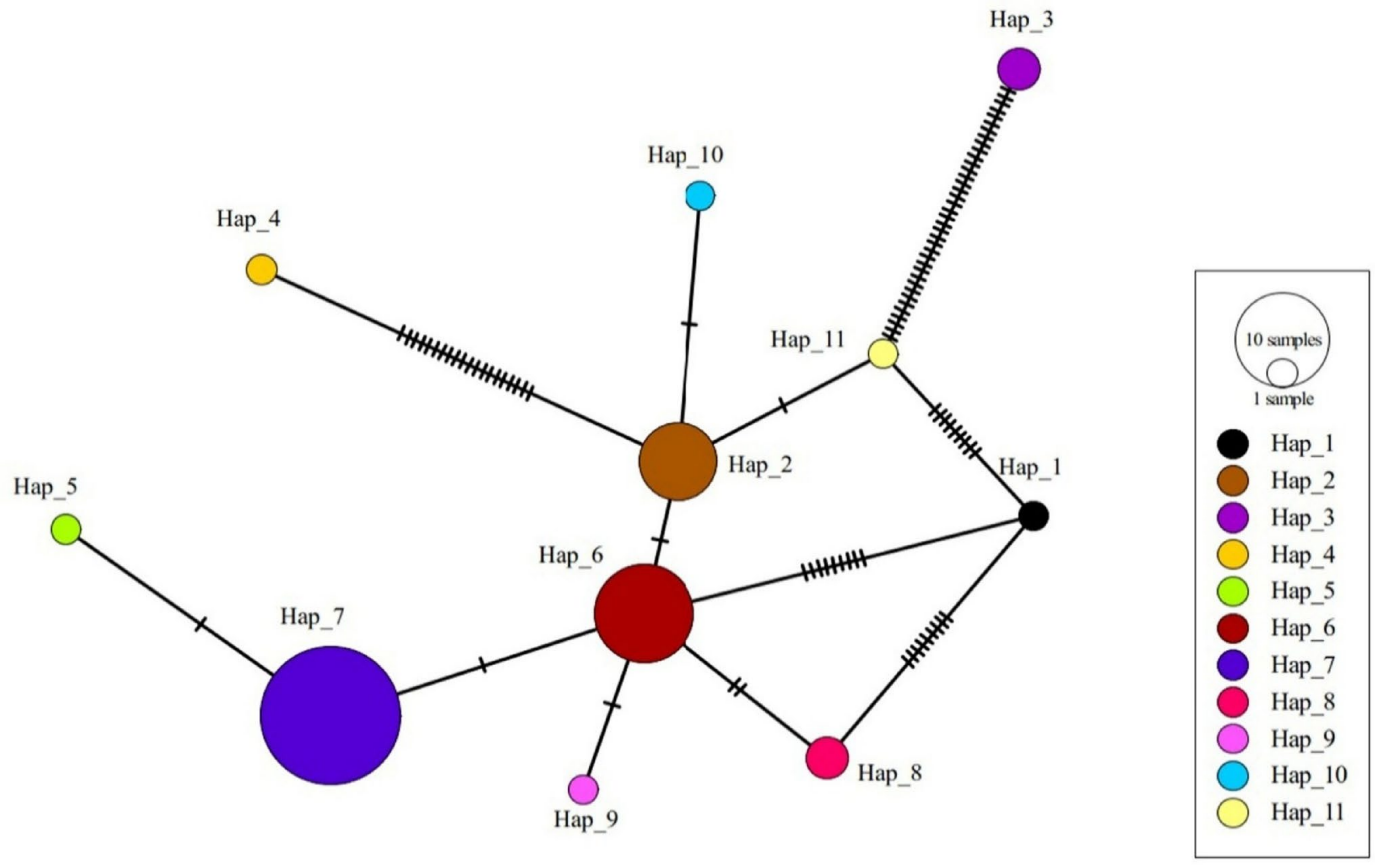
Haplotype network for the *COI* gene of *D. dendriticum* isolates. The diameter of each circle is proportional to the corresponding haplotype frequency. The lines linking the circles show the single mutation steps between haplotypes; each line on the graph shows a nucleotide substitution. Hap_1 is the *D. dendriticum* isolate identified in the study

*Dicrocoelium dendriticum* was the only trematode species identified in this study. It was detected in 3.67% of animals. DNA sequence analysis of the *D. dendriticum* isolate was performed, and the obtained nucleotide sequence was deposited to GenBank under accession number: PX706292. The 89.27–97.71% nucleotide similarities were seen between *D. dendriticum* isolate identified in this study and *D. dendriticum* isolates identified in Iran from goat (GenBank accession number: KX781720) and sheep (GenBank accession number: KX827427), China from sheep (GenBank accession number: KC164177) and goat (GenBank accession number: NC_025280), and in Japan from Sika deer (GenBank accession number: LC333984). The phylogenetic analyses of *D. dendriticum* isolates revealed that single-nucleotide polymorphisms (SNPs) were present in several parts of the *COI* gene regions. Genetic analyses of 50 *D. dendriticum COI* gene sequences present in the GenBank, including the isolate identified in the present study, revealed the presence of 11 *D. dendriticum* haplotypes circulating among hosts. Haplotype diversity was calculated as Hd: 0.74776, whereas nucleotide diversity was determined to be π = 0.01504. Other data about genetic analyses were listed in Table 3. Detailed information about the haplotype (haplotype name, GenBank accession numbers of isolates, animal species identified in isolates, geographic locations of isolates) is given in Supplementary Table 1. The networks of *D. dendriticum* haplotypes are shown in Fig. 2.

**Table 3:** Detailed information about genetic analyses of *D. dendriticum* and *P. ambiguus*

Species	Gene	*n*	H	Hd	π	Κt	Tajima’s D	FLF	FLD
*D. dendriticum*	*COI*	50	11	0.74776	0.01504	5.35265	− 2.20782*	− 1.51460*	− 0.91834*
*P. ambiguus*	*COI*	22	5	0.33766	0.00637	1.36364	− 2.41071*	− 3.77780*	− 3.60902*

n: number of isolates, H: number of haplotypes, Hd: Haplotype diversity, π: nucleotide diversity, Kt: Average number of nucleotide differences, *FLF*: Fu and Li’s F test statistic, *FLD*: Fu and Li’s D test statistic, Statistical significance: *, *P* < 0.01

*Andrya rhopalocephala* was the most common cestode species identified in this work, with a prevalence of 11.01%. DNA sequence analysis was performed on one of the detected *A. rhopalocephala* sample, and the consensus sequence determined was uploaded to GenBank under accession number: PX706293. The BLASTn analyses revealed that our *A. rhopalocephala* isolate had 97.19% nucleotide similarity with the *A. rhopalocephala* (GenBank accession number: AY189958) isolate identified in the European brown hare. In addition, our isolate had 88.47% nucleotide identity with *A. cuniculi*, which is another species that circulates among the European rabbit population (GenBank accession number: AY189957). Since there is only one sample of the nucleotide sequence of the *COI* gene region of *A. rhopalocephala* in GenBank, further genetic analyses could not be performed. However, the relatively low nucleotide similarity and high number of SNPs between the *A. rhopalocephala* isolate identified in this study and other *A. rhopalocephala* (GenBank accession number: AY189958) isolate suggests that they may be different genotypes.

*Mosgovoyia pectinata* (syn. *Cittotaenia pectinata*) was identified in 1.83% of the animals. DNA sequence analysis of the *M. pectinata* was also performed, and the obtained nucleotide data were deposited to GenBank under accession number: PX706296. With this study, nucleotide sequences belonging to the *COI* gene region of *M. pectinata* have been uploaded to GenBank. BLASTn analyses revealed that our *M. pectinata* isolate showed 93.29–94.01% nucleotide similarity with *M. pectinata* isolates detected in *Lepus timidus* from Kazakhstan (GenBank accession numbers: PV855722 and PV855723), Denmark (GenBank accession number: OQ421548), and the Faroe Islands (GenBank accession number: ON754342). Numerous SNPs were seen at multiple points on the *COI* gene between the *M. pectinata* isolates identified in this study and the *M. pectinata* isolates present in the GenBank (data not shown).

*Trichuris leporis* was the most prevalent helminth species identified in this study, with prevalence of 52.29%. DNA obtained from *T. leporis* was subjected to PCR using JB3-JB4.5 primers, but no PCR product was obtained with the primers for DNA sequence analysis. Therefore, phylogenetic analyses of *T. leporis* could not be performed.

**Fig. 3:**
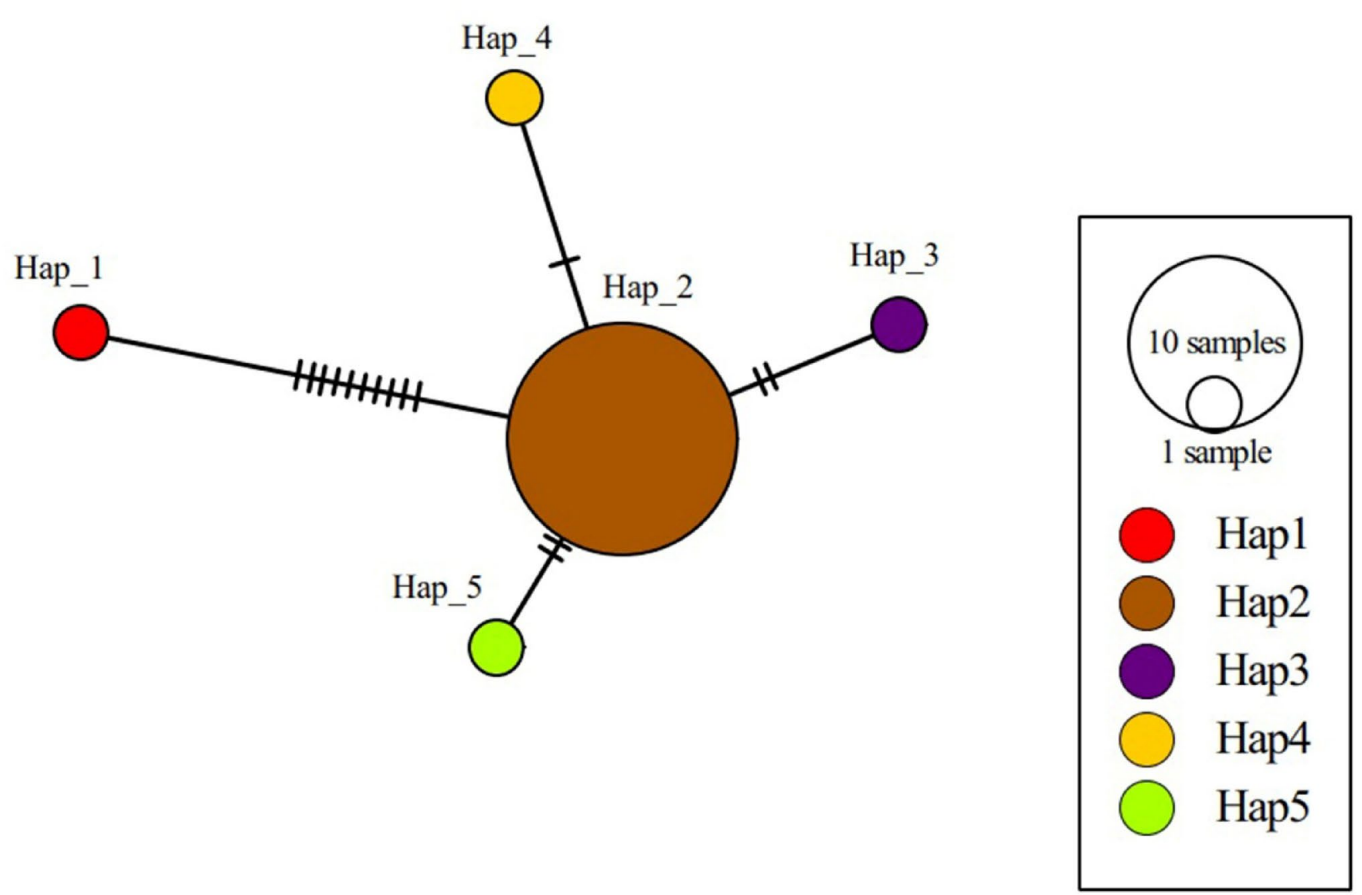
Haplotype network for the *COI* gene of *P. ambiguus* isolates. The diameter of each circle is proportional to the corresponding haplotype frequency. The lines linking the circles show the single mutation steps between haplotypes; each line on the graph shows a nucleotide substitution. Hap_1 is the *P. ambiguus* isolate identified in the study

*Passalurus ambiguus* was the second most common helminth species, with a prevalence of 31.19%. DNA sequence analysis was performed on a randomly selected sample, and the resulting sequence was registered in GenBank with the accession number PX706294. BLASTn analyses showed that our *P. ambiguus* isolate had 88.89–96.21% nucleotide similarity with *P. ambiguus* isolates reported in rabbit from China (GenBank accession numbers: KF472053-KF472072) and Egypt (GenBank accession number: MG991568). The phylogenetic analyses of the *COI* gene regions revealed that numerous SNPs and insertion/deletion (in/del) mutations were present between isolates (data not shown). Genetic analyses of 22 *P. ambiguus COI* gene sequences present in the GenBank, including the isolate detected in the study, demonstrated the presence of five *P. ambiguus* haplotypes circulating among hosts. Haplotype diversity was calculated as Hd: 0.33766, whereas nucleotide diversity was determined to be π = 0.00637. Other data about genetic analyses were listed in Table 3. Detailed information about the haplotype (haplotype name, GenBank accession numbers of isolates, host animal species from which the isolates were obtained, geographic locations of isolates) is given in Supplementary Table 2. Haplotype networks of *P. ambiguus* are shown in Fig. 3.

*Nematodirus leporis* was found in 22.94% of the animals. Subsequently, DNA sequence analysis was performed on one sample, and the obtained consensus sequence was deposited in GenBank with the accession number PX706295. The nucleotide sequences of the partial region *N. leporis COI* gene have been uploaded to GenBank. These are the first nucleotide data on *N. leporis* in the GenBank. Further phylogenetic analyses could not be performed because no nucleotide sequence for the *COI* gene region of this nematode was found in GenBank. However, BLASTn analyses also revealed that our *N. leporis COI* gene sequence shared 87.96% nucleotide similarity with *Nematodirus spathiger* (GenBank accession number: NC_024638), a species occurring in ruminants.

## Discussion

The European brown hare is an important member of healthy terrestrial ecosystems. Studies conducted in recent years indicate that the number of these animals has declined due to the expansion of agricultural areas, increased hunting activities, and diseases [2–5]. Parasitic pathogens cause serious clinical manifestations and even death in the population of this species [13, 15]. The number of studies conducted in Türkiye to investigate helminth species in the European brown hare is quite low [17, 19–21]. No data are present about the genetic features of helminth species circulating in this animal.

Numerous studies have been conducted in various parts of Europe to investigate the presence and prevalence of helminth species in the European brown hare. These studies have shown that the prevalence of helminth species in this host species varies between 54.5% and 100% [12, 14, 15, 39–42]. In the present study, 70.64% (77/109) of the animals were infected with at least one helminth species. Studies have also revealed that mixed infections involving multiple helminth species are quite common in the European brown hare [10, 12, 14, 15, 41]. Furthermore, the infection rate with one helminth species was 35.78% (39/109), whereas the mixed infection rate was 34.86% (38/109). It has been documented that infection with multiple helminth species can lead to severe clinical symptoms and death of hares, which may cause significant declines in the European brown hare population [10, 11, 13, 16]. Therefore, it is considered that identifying the helminth species circulating in European brown hares will contribute to the conservation of this animal populations.

*Dicrocoelium dendriticum* is one of the most common trematode species and is distributed worldwide with the exception of South Africa and Australia [26, 31]. The final hosts of *D. dendriticum* are mostly ruminants, but it can also infect rabbits, pigs, horses, dogs, rodents and humans. The first intermediate hosts of this trematode are snails (*Cionella* spp. and *Zebrina* spp.) and the second intermediate hosts are ants (*Formica* spp., frequently *F. fusca*) [31]. The European brown hare is one of the final hosts of *D. dendriticum*, and this trematode has been identified in Germany [43], Italy [14, 16], Greece [13], Bulgaria [44, 45], Romania [15], Belarus [41], and Türkiye [17, 21] in this animal. In this work, *D. dendriticum* is the only trematode species identified, with a prevalence of 3.67%. The BLASTn analyses of partial parts of the *COI* gene region revealed that 89.22–97.71% nucleotide similarities were present between our *D. dendriticum* isolate and *D. dendriticum* isolates present in the GenBank. Phylogenetic analyses revealed that SNPs were observed in many regions of the *COI* gene of *D. dendriticum* isolates identified in various countries (data not shown). Further genetic analyses of 50 *D. dendriticum COI* gene regions obtained from several hosts also determined that 11 *D. dendriticum* haplotypes are circulating in different parts of the world. Based on the genetic diversity data of *D. dendriticum COI* gene region, it is thought that there is moderate haplotype diversity (Hd: 0.74776) within the population, that the nucleotide diversity (π: 0.01504) between haplotypes is also significant, and that the negative neutrality test results may be related to population expansion (Table 3). However, it is considered that *D. dendriticum* isolates obtained from larger areas and different host species (both intermediate hosts and other final hosts such as cattle, horses, and buffaloes, etc.) are needed to obtain more detailed genetic analyses data.

*Andrya rhopalocephala* is a cestode species that infects the small intestine of hosts, and this parasite has a complex life cycle. This cestode is a rarely reported parasite in the European brown hare populations [32]. *Andrya rhopalocephala* has been reported in Poland by Czaplińska [39], Germany by Nickel and Gottwald [43], Bulgaria by Wickström et al. [46], and Austria and the Czech Republic by Chroust et al. [47]. In this study, 11.01% of the animals were found to be infected with *A. rhopalocephala.* This species was identified for the first time in Türkiye with this study. DNA sequence analysis of the *COI* gene of *A. rhopalocephala* was performed, and 97.19% nucleotide similarity was seen between our *A. rhopalocephala* isolate and *A. rhopalocephala* isolate present in the GenBank. The relatively low nucleotide similarity between our *A. rhopalocephala* isolate identified in this study and other *A. rhopalocephala* isolate (GenBank accession number: AY189958), along with the presence of numerous SNPs in the *COI* gene region (data not shown), suggests that multiple genotypes are circulating in the European brown hare population. In this study, further genetic analyses could not be performed due to insufficient nucleotide sequence data available in GenBank for *A. rhopalocephala* isolates. However, it is considered that comprehensive phylogenetic studies are needed to better understand genetic differentiations between *A. rhopalocephala* isolates.

*Mosgovoyia pectinata* has an indirect life cycle, and oribatid mites act as intermediate hosts for this parasite [31, 48]. *M. pectinata* is one of the most prevalent cestode species circulating among the European brown hare population, and this species was reported in England [40], Austria and Czechia [47], Poland [39], Belarus [41], Bulgaria [44], and Finland [49]. *M. pectinata* was first identified in Türkiye in 1988 [18], and this cestode has not been found in the following studies [20, 21]. In this study, 1.83% of the animals were found to be infected with *M. pectinata*. DNA sequence analyses of *M. pectinata COI* gene was done, and 93.29–94.01% nucleotide identities were seen between our *M. pectinata* isolate and other *M. pectinata* isolates present in the GenBank. It is considered that the low nucleotide similarity among *M. pectinata* isolates may be related to the different hosts in which the cestode was detected. While the *M. pectinata* isolate identified in the study was obtained from *Lepus europaeus*, the other isolates were obtained from *Lepus timidus*. These results indicate that extensive molecular studies are needed to better understand the genetic relationships of *M. pectinata* isolates circulating in different hosts.

*Trichuris leporis*, commonly known as whipworm, has a direct life cycle and infects the large intestines of its hosts [31]. *Trichuris leporis* larvae that molt in the intestinal mucosa can cause disorders such as anemia, digestive system problems, and deterioration of general health in hosts. Besides the above-mentioned effects, metabolic toxins produced by *T. leporis* may cause necrotic lesions inside the gut wall [7]. This nematode has been identified in various parts of the world, such as Austria [47], Germany [43], Italy [42], Greece [13], Slovakia [12], the Czech Republic [11, 47], Poland [50], Moldova [51], and Kazakhstan [52], with prevalence rates ranging from 1.9 to 55.41%. In this study, *T. leporis* was the most prevalent helminth species, and the infection rate was 52.29%. This nematode was identified in Türkiye by Merdivenci [17], Taşan [19], and Gurler and Doğanay [21], with the prevalence of 1.82-2%. The variation in the prevalence of *T. leporis* in studies may be related to the climate and environmental conditions of the study area and the number of animals included in the study. Although *T. leporis* is known to have a low pathogenic effect, it should be noted that in cases of heavy parasitemia, it can cause severe clinical symptoms in leverets and lead to significant declines in the populations [7, 9].

*Passalurus ambiguus*, which has a direct life cycle, parasitizes the large intestine of its hosts [31, 53]. The larval development of *P. ambiguus* occurs at the anal margin of its hosts, and transmission takes place through contact between hosts, coprophagy, or ingestion during grooming [53]. *Passalurus ambiguus* is normally considered a non-pathogenic helminth, but it has been determined that it can cause deaths in young animals [9, 54]. This nematode has been detected among the European brown hare [15, 41, 42, 50–52]; however, researchers claimed that *P. ambiguus* was more prevalent in the European rabbit than the European brown hare [9]. This nematode was the second most prevalent helminth species identified in this study, with a prevalence of 31.19%. Moreover, *P. ambiguus* was found to be the species with the highest mean intensity and mean abundance compared to the other parasites identified in this study (Table 1). This study provides the first DNA sequence data of the *P. ambiguus COI* gene region in Türkiye. The obtained nucleotide sequences were compared with *P. ambiguus* isolates in GenBank, revealing a nucleotide similarity of 88.89–96.21% among the isolates. Further genetic analyses of *P. ambiguus* isolates revealed that moderate number of haplotypes (H: 5), low haplotype diversity (Hd: 0.33776), and low to moderate nucleotide diversity (π: 0.00637). The fact that all Tajima’s D, FLF, and FLD tests showed negative and significant results can be interpreted as indicating population expansion, an excess of low-frequency mutations, and/or the occurrence of purifying selection (Table 3). However, since only 22 *P. ambiguus COI* gene sequences could be included in the study, it is considered that these interpretations need to be supported by more comprehensive genetic studies.

*Nematodirus leporis* has a direct life cycle, and infection occurs through the ingestion of infective-stage larvae [31]. In severe infections caused by this nematode in domestic animals, diarrhea and weight loss have been observed. It can also cause atrophy, degeneration, and necrosis in the intestinal villi [31]. *Nematodirus leporis* is one of the species rarely detected in the European brown hares; this nematode was reported in Türkiye [20, 21] and Kazakhstan [52]. In this study, *N. leporis* was identified in 22.94% of the animals. This study is the first to reveal partial nucleotide sequences of the *COI* gene region of *N. leporis* in Türkiye, and the data were uploaded to GenBank. It is thought that these nucleotide data will serve as a resource for future studies. Although the data obtained from the study are considered important, extensive studies are needed to determine the genetic characteristics of *N. leporis* using nucleotide data from different gene regions. Additionally, Durette-Desset proposed that *Nematodirus* species detected in lagomorphs are to be classified in the genus *Rauschia* [55]. It is considered that comparative morphological and genetic analyses of *Nematodirus* species obtained from both ruminants and lagomorphs are needed to confirm this classification.

Studies have shown that the distribution of helminth species might be affected by several factors, such as individual factors of hosts (age, sex, immune status, etc.), population factors (density, migrations, etc.), and environmental conditions (habitat) [10, 53]. Moreover, it has also been observed that species that require intermediate hosts to complete their life cycles can be more affected by environmental factors and that their prevalence among animals may decrease, particularly in regions with harsh climatic conditions [10, 56]. In this study, the most prevalent helminth species were *T. leporis* (52.29%), *P. ambiguus* (31.19%), and *N. leporis* (22.94%), and all of these species have a direct life cycle [31, 53]. Moreover, the mean intensity and abundance of these species were higher than those of the other helminth species identified in this study (Table 1). Similar results were also observed in studies conducted in several countries [10, 12, 13, 15, 57]. Researchers argued that the prevalence of species with direct life cycles compared to those with indirect life cycles may be related to environmental and climatic conditions in the study area, as these conditions could affect the abundance of suitable intermediate hosts in the sampling area [57]. Similarly, it is known that the harsh continental climate conditions of Sivas province, where the study was conducted, are not favorable to increasing the population density of intermediate hosts of certain helminth species. Therefore, in this study, it is thought that the prevalence of helminth species with a direct life cycle is higher than that of helminths with an indirect life cycle.

In conclusion, internal organs belonging to 109 European brown hares were researched for helminths using microscopic and molecular methods in this work. Six helminths, *Dicrocoelium dendriticum*, *Andrya rhopalocephala*,* Mosgovoyia pectinata*,* Trichuris leporis*,* Passalurus ambiguus*, and *Nematodirus leporis*, were identified in liver and intestinal samples. No helminth was detected in spleen, lung, heart, and kidney samples. *Andrya rhopalocephala* was detected for the first time with this study in Türkiye. Moreover, phylogenetic analyses of identified helminths, except for *Trichuris leporis*, were done. It is considered that the obtained data help to understand epidemiology and genetic features of helminth species. However, it is considered that more extensive studies are needed to understand the presence and prevalence of parasites circulating in the European brown hare population in Türkiye and their genetic characteristics.

## Supplementary Information

Below is the link to the electronic supplementary material.

Supplementary Material 1

## Data Availability

All data generated or analyzed during this study are included in this manuscript.

## References

[1] Bock A (2020) Lepus europaeus (Lagomorpha: Leporidae). Mamm Species 52:125–142

[2] Hackländer K, Schai-Braun S (2019) Lepus europaeus. IUCN Red List Threatened Species eT41280A45187424. 10.2305/IUCN.UK.2019-1.RLTS.T41280A45187424.en

[3] Smith RK, Vaughan Jennings N, Harris S (2005) Abundance and demography of European hares. Mamm Rev 35(1):1–24. 10.1111/j.1365-2907.2005.00057.x

[4] Santilli F, Galardi L (2016) Habitat structure and farming effects on European hare abundance. Hystrix 27:120

[5] Pavliska PL, Riegert J, Grill S, Šálek M (2018) Landscape heterogeneity and population density of European hare. Mamm Biol 88:8–15. 10.1016/j.mambio.2017.11.003

[6] Tsokana CN, Sokos C, Giannakopoulos A, Birtsas P, Valiakos G, Spyrou V, Athanasiou LV, Burriel AR (2020) European brown hare as a source of emerging pathogens. Vet Med Sci 6:550–564. 10.1002/vms3.248 32088933 10.1002/vms3.248 PMC7397891

[7] Wibbelt G, Frölich K (2005) Infectious diseases in European brown hare. Wildl Biol Pract 86–93. 10.2461/wbp.2005.1.11

[8] Bełkot Z, Wojtas N, Chmielowiec A, Kowalczyk J, Drozd Ł (2024) Infectious and parasitic diseases in European hares (*Lepus europaeus* Pall.) from the Lublin Upland detected by macroscopic autopsy. Medycyna Wet 80:519–526. 10.21521/mw.6942

[9] Hughes K (2024) Endoparasites of rabbits and hares. J Vet Diagn Invest 36:599–616. 10.1177/10406387241261991 39108102 10.1177/10406387241261991 PMC11459662

[10] Alzaga V, Tizzani P, Acevedo P, Ruiz-Fons F, Vicente J, Gortázar C (2009) Deviance partitioning of host factors affecting parasitization in the European brown hare (*Lepus europaeus*). Naturwissenschaften 96:1157–1168. 10.1007/s00114-009-0577-y 19565211 10.1007/s00114-009-0577-y

[11] Lukešová D, Langrová I, Vadlejch J, Jankovská I, Hlava J, Válek P, Čadková Z (2012) Endoparasites in European hares under gamekeeping conditions. Helminthologia 49:159–163. 10.2478/s11687-012-0032-z

[12] Dubinský P, Vasilková Z, Hurníková Z, Miterpáková M, Slamečka J, Jurčík R (2010) Parasitic infections of the European brown hare (*Lepus europaeus*) in south-western Slovakia. Helminthologia 47:219–225. 10.2478/s11687-010-0034-7

[13] Diakou A, Sokos C, Papadopoulos E (2014) Endoparasites found in European brown hares (*Lepus europaeus*) hunted in Macedonia. Greece Helminthologia 51:345–351. 10.2478/s11687-014-0251-6

[14] Sergi V, Romeo G, Serafini M, Torretta E, Macchioni F (2018) Endoparasites of the European hare in central Italy. Helminthologia 55:127–134. 10.2478/helm-2018-0011 31662638 10.2478/helm-2018-0011 PMC6799548

[15] Ana-Maria JSB, Bogdan SC, Tiana F, Ionela H, Gheorghe D (2022) Endoparasites found in *Lepus europaeus* hunted in western Romania. Int Multidiscip Sci GeoConf SGEM 22:691–697

[16] Brustenga L, Franciosini MP, Diaferia M, Rigamonti G, Musa L, Russomanno BL, Veronesi F (2023) Parasitological survey in European brown hare (*Lepus europaeus*) breeding facilities in southern Italy. Pathogens 12:208. 10.3390/pathogens12020208 36839480 10.3390/pathogens12020208 PMC9964568

[17] Merdivenci A (1983) Türkiye’de ilk kez bildirilen parazitler (1952–1982). Türk Mikrobiyol Cem Derg 13:23–38

[18] Taşan E (1988) Türkiye’de yabani tavşanlarda ilk *Mosgovia pectinata* bulgusu. Ankara Üniv Vet Fak Derg 35:540–544

[19] Taşan E (1989) Elazığ ve Tunceli yörelerinde yabani tavşanların helmintleri. Fırat Üniv Sağlık Bil Derg 3:75–81

[20] Doğanay A, Gürler AT (2007) Yaban tavşanında *Nematodirus leporis* olgusu. Ankara Üniv Vet Fak Derg 54:141–143. 10.1501/Vetfak_0000000269

[21] Gürler AT, Doğanay A (2007) Ankara ve civarında bulunan tavşanlarda solunum ve sindirim sistemi helmintlerinin yaygınlığı. Ankara Üniv Vet Fak Derg 54:105–109. 10.1501/Vetfak_0000000259

[22] Ergün A, Buldur AD (2016) Sivas ilinde yükselti basamaklarına göre 1990–2015 yılları arasında nüfus ve yerleşmelerin dağılışı. ZfWT 8:303–327

[23] Kartal F (2024) Sivas ili topografya ve bazı arazi özelliklerinin CBS ile incelenmesi. ISPEC Int J Soc Sci Humanit 8:152–169

[24] Asan H, Yalçın HM, Şimşek E (2018) Sivas ili kuş gözlem turizmi potansiyelinin değerlendirilmesi. ASOS Araşt Derg 6:630–655. 10.16992/ASOS.13859

[25] Hofer S, Gloor S, Müller U, Mathis A, Hegglin D, Deplazes P (2000) High prevalence of *Echinococcus multilocularis* in urban red foxes. Parasitol 120:135–142. 10.1017/s0031182099005351 10.1017/s0031182099005351 10726275

[26] Bowman DD (2014) Diagnostic parasitology. 10th ed. In: Bowman DD (Ed). Georgis’ Parasitology for Veterinarians. St. Louis: Elsevier

[27] Mönnig HO (1950) Veterinary helminthology and entomology, 3rd edn. Baillière, Tindall and Cox, London

[28] Beveridge I (1978) A taxonomic revision of the genera Cittotaenia Riehm, 1881, Ctenotaenia Railliet, 1893, Mosgovoyia Spasskii, 1951 and Pseudocittotaenia Tenora, 1976 (Cestoda: Anoplocephalidae). Mémoires du Muséum Natl D’Histoire Naturelle Nouvelle Série Série Zool 107:1–64

[29] Anderson RC (2000) Nematode parasites of vertebrates: their development and transmission, 2nd edn. CABI Publishing, Wallingford

[30] Tenora F, Koubková B, Feliu C (2002) Redescription of *Andrya cuniculi*. Folia Parasitol 49:50–54. 10.14411/fp.2002.011 11993551 10.14411/fp.2002.011

[31] Taylor MA, Coop RL, Wall RL (2016) Veterinary parasitology, 4th edn. Blackwell Publishing, Oxford

[32] Haukisalmi V (2023) *Andryoides* gen. n. and morphological key features in cestodes of the family Anoplocephalidae. Folia Parasitol 70:1–10. 10.14411/fp.2023.006 10.14411/fp.2023.006 36999366

[33] Bowles J, Blair D, McManus DP (1992) Genetic variants within the genus *Echinococcus* identified by mitochondrial DNA sequencing. Mol Biochem Parasitol 54:165–173. 10.1016/0166-6851(92)90109-w 1435857 10.1016/0166-6851(92)90109-w

[34] Erol U, Sarimehmetoglu O, Utuk AE (2021) Intestinal system helminths of red foxes and molecular characterization of Taeniid cestodes. Parasitol Res 120:2847–2854. 10.1007/s00436-021-07227-3 34232387 10.1007/s00436-021-07227-3

[35] Tamura K, Stecher G, Kumar S (2021) MEGA11: molecular evolutionary genetics analysis. Mol Biol Evol 38:3022–3027. 10.1093/molbev/msab120 33892491 10.1093/molbev/msab120 PMC8233496

[36] Rozas J, Ferrer-Mata A, Sánchez-DelBarrio JC, Guirao-Rico S, Librado P, Ramos-Onsins SE, Sánchez-Gracia A (2017) DnaSP 6: DNA sequence polymorphism analysis of large data sets. Mol Biol Evol 34:3299–3302. 10.1093/molbev/msx248 29029172 10.1093/molbev/msx248

[37] Bandelt HJ, Forster P, Röhl A (1999) Median-joining networks for inferring intraspecific phylogenies. Mol Biol Evol 16:37–48. 10.1093/oxfordjournals.molbev.a026036 10331250 10.1093/oxfordjournals.molbev.a026036

[38] Leigh JW, Bryant D (2015) POPART: full-feature software for haplotype network construction. Methods Ecol Evol 6(9). 10.1111/2041-210X.12410

[39] Czaplińska D, Czapliński B, Rutkowska M (1965) Studies on the European hare. IX. Helminth fauna in the annual cycle. Acta Theriol 10:55–78

[40] Irvin AD (1970) Gastro-intestinal parasites of British hares. J Zool 162:544–546

[41] Shimalov VV (2001) Helminth fauna of the hare in Belarus. Parasitol Res 87:85. 10.1007/s004360000259 11199857 10.1007/s004360000259

[42] Catalano S, La Morgia V, Molinar Min AR, Fanelli A, Meneguz PG, Tizzani P (2022) Gastrointestinal parasite community and phenotypic plasticity in native and introduced alien Lagomorpha. Animals 12:1287. 10.3390/ani12101287 35625133 10.3390/ani12101287 PMC9138120

[43] Nickel S, Gottwald A (1979) Endoparasites of the hare (*Lepus europaeus*). Angew Parasitol 20:57–62 507443

[44] Panayotova-Pencheva MS (2022) Endoparasites of the European brown hare from Bulgaria. Ann Parasitol 68:553–562. 10.17420/ap6803.462 36587612 10.17420/ap6803.462

[45] Trifonova AP, Panayotova-Pencheva MS, Kaneva EM (2023) Infections with *Dicrocoelium dendriticum* and others in European brown hares. Bulgarian J Vet Med 26(2). 10.15547/bjvm.2021-0025

[46] Wickström LM, Haukisalmi V, Varis S, Hantula J, Henttonen H (2005) Molecular phylogeny of anoplocephaline cestodes. Syst Parasitol 62:83–99. 10.1007/s11230-005-5488-5 16167118 10.1007/s11230-005-5488-5

[47] Chroust K, Vodnansky M, Pikula J (2012) Parasite load of European brown hares in Austria and the Czech Republic. Vet Med 57. 10.17221/6367-VETMED

[48] Stunkard HW (1941) Life history of anoplocephaline cestodes of hares. J Parasitol 27:299–325

[49] Soveri T, Valtonern M (1983) Endoparasites of hares (*Lepus timidus* L. and *L. europaeus* Pallas) in Finland. J Wildl Dis 19:337–341. 10.7589/0090-3558-19.4.337 6644932 10.7589/0090-3558-19.4.337

[50] Kornaś S, Wierzbowska I, Wajdzik M, Kowal J, Basiaga M, Nosal P (2014) Endoparasites of European brown hare from southern Poland. Ann Anim Sci 14:297–305. 10.2478/aoas-2014-0010

[51] Rusu Ş (2020) Parasitic fauna of hare from Codrii natural reservation. Sci Pap Vet Med 2:108–114

[52] Kiyan V, Smagulova A, Manapov N, Jazina K, Uakhit R, Bulashev A, Lider L, Leontyev S (2025) Parasitic fauna of *Lepus europaeus* and *Lepus timidus* in Kazakhstan. Biology 14:1083. 10.3390/biology14081083 40906407 10.3390/biology14081083 PMC12383461

[53] Boag B, Lello J, Fenton A, Tompkins DM, Hudson PJ (2001) Patterns of parasite aggregation in the wild European rabbit (*Oryctolagus cuniculus*). Int J Parasitol 31:1421–1428. 10.1016/s0020-7519(01)00270-3 11595228 10.1016/s0020-7519(01)00270-3

[54] Rinaldi L, Russo T, Schioppi M, Pennacchio S, Cringoli G (2007) *Passalurus ambiguus*: copromicroscopic diagnosis. Parasitol Res 101:557–561. 10.1007/s00436-007-0513-z 17372763 10.1007/s00436-007-0513-z

[55] Durette-Desset MC (1979) Les Nematodirinae (Nematoda) chez les ruminants et chez les lagomorphes. Ann Parasitol Hum Comp 54:313–329 525956

[56] Arneberg P (2002) Host population density and body mass as determinants of species richness in parasite communities: comparative analyses of directly transmitted nematodes of mammals. Ecography 25:88–94. 10.1034/j.1600-0587.2002.250110.x

[57] Bordes F, Langand J, Feliu C, Morand S (2007) Helminth communities of an introduced hare (*Lepus granatensis*) and a native hare (*Lepus europaeus*) in southern France. J Wildl Dis 43:747–751. 10.7589/0090-3558-43.4.747 17984274 10.7589/0090-3558-43.4.747

