# Supplementary material for: Helminthological Survey of the European Brown Hare (Lepus europaeus) in Türkiye, with A Note on Haplotype and Network Analyses of Dicrocoelium dendriticum and Passalurus ambiguus
Source: Acta Parasitol. 2026 Apr 21;71(3):84. doi: 10.1007/s11686-026-01278-1 (PMC13099703; doi:10.1007/s11686-026-01278-1)
Supplement: Supplementary file 1 — Supplementary Material 1 [file 11686_2026_1278_MOESM1_ESM.docx]

**Supplementary Table 1.** Detailed information about *D. dendriticum* haplotypes.

| **Haplotye**  **name** | **Number of isolates** | **GenBank accession number** | **Animal species identified helminth** | **Country** |
| --- | --- | --- | --- | --- |
| Haplotype-1 | 1 | PX706292 | European Brown hare | Türkiye |
| Haplotype-2 | 7 | KX827444 | Sheep | Iran |
|  |  | KX827443 |  |  |
|  |  | KX827442 |  |  |
|  |  | KX827440 |  |  |
|  |  | KX827437 |  |  |
|  |  | KX827433 |  |  |
|  |  | KC164177 | Sheep | China |
| Haplotype-3 | 2 | KF318787 | Goat | China |
|  |  | NC025280 |  |  |
| Haplotype-4 | 1 | LC333984 | Sika deer | Japan |
| Haplotype-5 | 1 | LC333985 | Sika deer | Japan |
| Haplotype-6 | 11 | KC164176 | Sheep | China |
|  |  | KX827439 | Sheep | Iran |
|  |  | KX827438 |  |  |
|  |  | KX827436 |  |  |
|  |  | KX827435 |  |  |
|  |  | KX827434 |  |  |
|  |  | KX827432 |  |  |
|  |  | KX827428 |  |  |
|  |  | KX827427 |  |  |
|  |  | KX781720 | Goat |  |
|  |  | KX781718 |  |  |
| Haplotype-7 | 22 | KC164175 | Sheep | China |
|  |  | KC164174 |  |  |
|  |  | KX827431 | Sheep | Iran |
|  |  | KX827429 |  |  |
|  |  | KC164195 | Goat | China |
|  |  | KC164194 |  |  |
|  |  | KC164193 |  |  |
|  |  | KC164192 |  |  |
|  |  | KC164191  KC164190 |  |  |
|  |  | KC164189  KC164188 |  |  |
|  |  | KC164187 |  |  |
|  |  | KC164186 |  |  |
|  |  | KC164185 |  |  |
|  |  | KC164184  KC164183 |  |  |
|  |  | KC164182 |  |  |
|  |  | KC164181 |  |  |
|  |  | KC164180 |  |  |
|  |  | KC164179 |  |  |
|  |  | KC164178 |  |  |
| Haplotype-8 | 2 | KX827441 | Sheep | Iran |
|  |  | KX781719 | Goat |  |
| Haplotype-9 | 1 | KX827430 | Sheep | Iran |
| Haplotype-10 | 1 | KX781722 | Goat | Iran |
| Haplotype-11 | 1 | KX781721 | Goat | Iran |

**Supplementary Table 2.** Detailed information on *P. ambiguus* haplotypes.

| **Haplotye**  **name** | **Number of isolates** | **GenBank accession number** | **Animal species identified helminth** | **Country** |
| --- | --- | --- | --- | --- |
| Haplotype-1 | 1 | PX706294 | European Brown hare | Türkiye |
| Haplotype-2 | 18 | MG991568 | Domestic rabbits | Egypt |
|  |  | KF472053  KF472054  KF472058  KF472059  KF472060  KF472061  KF472062  KF472063  KF472064  KF472065  KF472066  KF472067  KF472068  KF472069  KF472070  KF472071  KF472072 | Rabbit | China |
| Haplotype-3 | 1 | KF472057 | Rabbit | China |
| Haplotype-4 | 1 | KF472056 | Rabbit | China |
| Haplotype-5 | 1 | KF472055 | Rabbit | China |
